# School Refusal Behavior and Aggression in Spanish Adolescents

**DOI:** 10.3389/fpsyg.2021.669438

**Published:** 2021-04-29

**Authors:** Carolina Gonzálvez, Miriam Martín, María Vicent, Ricardo Sanmartín

**Affiliations:** Department of Development Psychology and Teaching, University of Alicante, Alicante, Spain

**Keywords:** school refusal behavior, physical aggression, verbal aggression, anger, hostility, adolescents

## Abstract

In order to reduce school attendance problems and aggressive behavior, it is essential to determine the relationship between both variables. The aim of this study was twofold: (1) to examine the mean differences in scores on aggression, based on school refusal behavior, and (2) to analyze the predictive capacity of high scores on aggression, based on school refusal behavior factors. The sample consisted of 1455 Spanish secondary school students, aged 13–17 (*M* = 14.85; SD = 1.56). The School Refusal Assessment Scale-Revised (I. Avoidance of negative affectivity, II. Escape from aversive social and/or evaluative situations, III. Pursuit of attention from significant others, and IV. Pursuit of tangible reinforcement outside of school) and the Aggression Questionnaire (I. Physical Aggression, II. Verbal Aggression, III. Anger, and IV. Hostility) were used. Results indicated that students having high levels of Physical Aggression, Verbal Aggression, Anger, and Hostility received significantly higher scores on school refusal behavior. In most cases, school refusal behavior was found to be a positive and statistically significant predictor of aggression. Students that base their school refusal on the pursuit of tangible reinforcements outside of school earned higher scores, and other functional conditions underlying school refusal behavior were found to be associated with aggression issues. The role of aggression as a risk factor for school refusal behavior is discussed.

## Introduction

**FIGURE 1:**
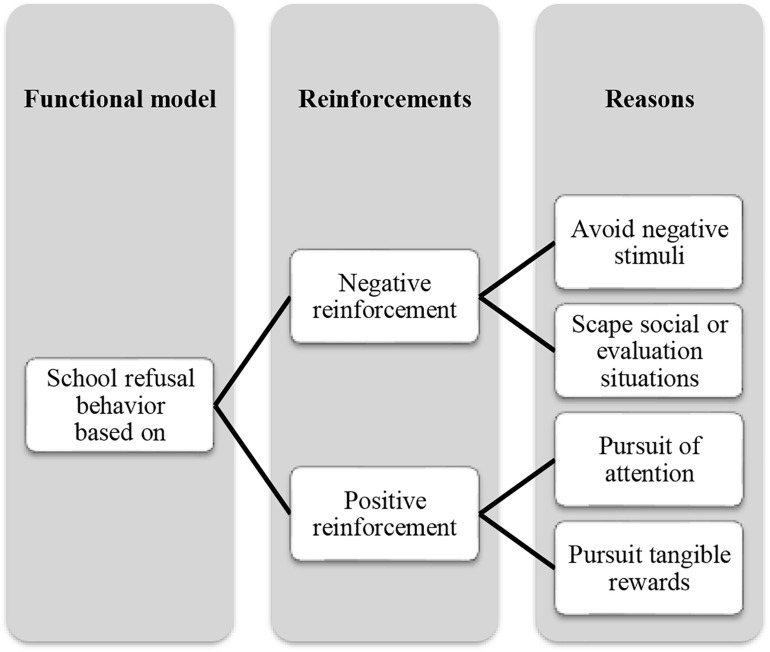
Functional model of school refusal behavior.

School attendance and academic success have long been recognized as fundamental and crucial competencies for children and adolescents (Kearney et al., 2019). However, school attendance problems concern educational authorities as they are considered a violation of school rules but also social norms (Donat et al., 2018). Problems regarding school attendance include distinct types of school absence or general difficulties in attending or staying in school (Heyne et al., 2019). It is difficult to establish one unique model that includes all of the potential causes of this behavior (Gonzálvez and Inglés, 2019). However, according to a functional model of school refusal behavior (see Figure 1), four basic conditions exist, upon which the failure to attend school may be based: I. Avoidance of stimuli that provoke negative affectivity, II. Escape from aversive social and/or evaluative situations, III. Pursuit of attention from significant others, and IV. Pursuit of tangible reinforcement outside of school (Kearney, 2002).

In a society that encourages the education of its youth, school attendance has become a major issue for political and educational authorities (Havik et al., 2014). Given that the social significance of attending school has been shown to favor the educational, social, and personal development of children, measures have been created to remedy school absenteeism, by enacting distinct legal provisions and prevention/intervention measures. An example of this is the Sustainable Development Goal 4 (SDG 4) of Agenda 2030, approved by the General Assembly of the United Nations in 2015, which urges governments to guarantee that all children complete their primary and secondary level education. In Spain, legal measures have been proposed to reduce school absenteeism, promoting early intervention for students. Specifically, article 8 of Decreto 104/2008 establishes the principles of equality and inclusion in Valencia’s educational system. It establishes plans to combat school absenteeism and early school dropout. School refusal demands early intervention to ensure the reincorporation of affected students as soon as possible. This helps avoid situations of inequality and social exclusion (Pehlivan, 2011; Ingul et al., 2019). Several familiar, personal, and social factors play a significant role in school refusal behavior; therefore, educational inclusion practices may improve students’ expectations and results. This, in turn, thus promotes their school adaptation (Fernández-Batanero, 2011). In this sense, the school plays a very important role, because depending on the quality of individual attention a student receives or the type of methodology used by the teacher, school refusal behavior could occur (Filippello et al., 2019).

Every year, approximately 246 million students experience some sort of violence in and around school (United Nations, 2017), with school attendance problems being one potential consequence of this violence (Shaikh et al., 2019). The individual’s response to school absenteeism plays a major role in their resulting emotions, which may range from intense excitement to anger or aggressiveness (Law et al., 2011). Our society has made a latent attempt to improve the quality of life of its citizens and to reduce violence. Therefore, an improved understanding of the influential factors underlying these types of aggressive behavior is necessary (Cupaioli et al., 2019). Baron and Byrne (2005) noted that the primary motives explaining aggression are social, personal, and situational factors. Furthermore, three basic components of aggressive behavior have been proposed: a cognitive component (hostility), an emotional component (anger), and a motor component (physical or verbal aggression) (Anderson and Bushman, 2002). It has been suggested that the main objective of physical aggression is to harm or humiliate (Ramírez and Andreu, 2003), and the objective of verbal aggression is to cause psychological harm (Piko and Keresztes, 2006). Anger is a response to threats that are real (or not real) (García et al., 2020), while hostility is a negative emotion resulting from refusal and that may lead to abusive and aggressive situations (Rendón, 2008).

Of the negative consequences of school absenteeism, evidence suggests that certain types of school refusal behaviors are linked to externalizing behavior problems (Vaughn et al., 2014). Cardwell et al. (2019) noted that a greater presence of risk factors (e.g., impulsiveness, poor relationship with parents, or antisocial behavior with peers) can lead to a greater probability of engaging in violent acts during adolescence. Previous studies have suggested that the transition into adolescence coincides with a period of psychological, biological, and emotional transformation (Martínez González and Álvarez Blanco, 2005), which may explain the increase in disruptive or aggressive behavior during this life phase (Buckley et al., 2012; Peltzer and Pengpid, 2017; Rocque et al., 2017). However, no studies with Spanish student samples have been found that analyze the relationship between school refusal (from a functional model) and aggression during this period. Only one study has been identified that examined the relationship between these variables, but in a sample of young children (Aparicio-Flores et al., 2020). In this study, it was determined that students with very aggressive behavior received higher scores on school refusal, and this refusal was a positive predictor of aggression. Differences based on sex and age were found in this study, highlighting the need for further research on the relationship between these variables during adolescence.

The objective of this study is to examine the relationship between school refusal and aggression in Spanish adolescents aged 13–17. This general objective has been narrowed down into two specific objectives: (a) to examine whether or not differences exist in mean scores on school refusal behavior in students with high and low scores on Physical Aggression, Verbal Aggression, Anger, and Hostility; and (b) to analyze the predictive capacity of school refusal on high scores in Physical Aggression, Verbal Aggression, Anger, and Hostility. Based on empirical evidence, the following hypotheses were proposed:

**Hypothesis 1:** It is expected that students with high levels of Physical Aggression, Verbal Aggression, Anger, and Hostility will score higher on school refusal behavior than their peers having low aggression levels (Echevarría and López-Zafra, 2011; Aparicio-Flores et al., 2020).
**Hypothesis 2:** It is expected that school refusal behavior will act as a positive and statistically significant predictor of high levels of Physical Aggression, Verbal Aggression, Anger, and Hostility (Wood et al., 2012; Agreda and Hinojo, 2015; Aparicio-Flores et al., 2020).

## Materials and Methods

### Participants

The initial sample consisted of 1518 students aged 13–17 (*M* = 14.85; SD = 1.56) (see Table 1). Of these participants, 63 were excluded because they either did not give the written informed consent from their parents (*N* = 37) or because there were errors or omissions in the completed questionnaires (*N* = 26). The final sample contained 1455 high school students (61% male). Sample selection was conducted via random cluster sampling (geographic areas: north, south, east, west, and center) in the province of Alicante (Spain), with the participation of 12 schools (eight public and four charter schools). The socio-economic level, based on the parents’ labor situation and academic education levels, was considered as middle class.

**TABLE 1:** Sample distribution across sex and age.

**Sex**	**Age**					**Total**
	**13**	**14**	**15**	**16**	**17**	
Boys	236	140	130	149	233	888
	16.2%	9.6%	8.9%	10.2%	16.0%	61.0%
Girls	199	116	70	73	109	567
	13.7%	8.0%	4.8%	5.0%	7.5%	39.0%
Total	435	256	200	222	342	1455
	29.9%	17.6%	13.7%	15.3%	23.5%	100%

### Instruments

School Refusal Assessment Scale-Revised (SRAS-R; Kearney, 2002). The SRAS-R is a self-reporting measure consisting of 24 items having a seven-point response scale (0 = never; 6 = always). This instrument assesses school refusal behavior in students aged 8–17. Specifically, it permits the assessment of causes of school absenteeism, using a functional model that proposes four factors that contribute to school refusal: I. Avoidance of stimuli that provoke negative affectivity (e.g., “How often do you stay away from school because if you go, you will feel sad or depressed?”), II. Escape from aversive social and/or evaluative situations (e.g., “If it were easier for you to make new friends, would it be easier for you to go to school?”), III. Pursuit of attention from significant others (e.g., “How much would you rather be taught by your parents at home than by your teacher at school?”), and IV. Pursuit of tangible reinforcement outside of school (e.g., “How often do you refuse to go to school because you want to have fun outside of school?”) (Kearney, 2002). In this study, the Spanish version of the SRAS-R developed by Gonzálvez et al. (2016) was used and the internal consistency was estimated by the Cronbach’s alpha coefficient. Optimal values were obtained in this study for each of the factors: 0.81 (Factor I), 0.80 (Factor II), 0.80 (Factor III), and 0.70 (Factor IV).

Aggression Questionnaire (AQ) (Buss and Perry, 1992). The AQ is a self-reporting measure consisting of 29 items that assess four components of aggression: I. Physical Aggression (nine items, e.g., “Given enough provocation, I may hit another person”), II. Verbal Aggression (five items, e.g., “I can’t help getting into arguments when people disagree with me”), III. Anger (seven items, e.g., “Sometimes I feel like a powder keg ready to explode”), and IV. Hostility (eight items, e.g., “When people are especially nice to me, I wonder what they want”). A five-point Likert scale was used for response to each item (1 = extremely uncharacteristic of me; 5 = extremely characteristic of me). In this study, the Spanish version of the AQ developed by Santisteban and Alvarado (2009) was used and acceptable reliability values estimated by Cronbach’s alpha were found for this instrument: 0.80 (Physical Aggression), 0.76 (Verbal Aggression), 0.71 (Anger), and 0.72 (Hostility).

### Procedure

First, an interview was conducted with the school management teams and informative letters were sent to all of the families, requesting their written informed consent for participation of the children. Once authorization was received, students anonymously and voluntarily completed the questionnaires. The measures were completed during school hours, in a 30-min session in which they were given all the pertinent instructions. In all of the sessions, at least one researcher was present, in addition to the classroom teacher. Finally, the members of the educational community were thanked for their collaboration. The Ethics Committee of the University of Alicante (code of ethics: UA-2017-09-05) approved the study and the standards established by the Declaration of Helsinki (Rickham, 1964) were followed.

### Statistical Analysis

To determine the differences between students with high aggression (scores equal to or higher than the 75th percentile) and low aggression (scores equal to and lower than the 25th percentile) on the mean scores on school refusal, the Student’s *t*-test was applied. In addition, the size of the differences was calculated using Cohen’s *d* (standardized difference between means) (Cohen, 1988). The *d* index was interpreted as follows: small size (0.20 ≤ *d* ≤ 0.50), medium size (0.51 ≤ *d* ≤ 0.79), and large size (*d* ≥ 0.80). The predictive capacity of school refusal on high scores on aggression was analyzed using the binary logistic regression method, with the forward stepwise procedure, in accordance with the Wald test. This predictive capacity was estimated using the OR (odds ratio). All of the analyses were carried out using the SPSS statistics program, version 25.0.

## Results

### Mean Differences

The differences found between the student groups with high and low scores on Physical Aggression, based on the type of school refusal behavior, are presented in Table 2. Results show that students having high scores on Physical Aggression obtained a higher mean score on school refusal behavior, with statistically significant differences for the first two factors of the SRAS-R (I. Avoidance of negative affectivity, II. Escape from aversive social and/or evaluative situations) and the fourth factor (IV. Pursuit tangible reinforcement outside of school). The size of the differences was found to be small for Factor II (*d* = 0.21) and Factor IV (*d* = 0.25), while it was medium for Factor I (*d* = 0.51).

**TABLE 2:** Differences in school refusal behavior in students with high and low scores on Physical Aggression.

**Levene’s test**			**Low score**		**High score**		**Statistics**			
**SRAS-R**	***F***	***p***	***M***	**SD**	***M***	**SD**	***t***	**d.f.**	***p***	***d***
Factor I	15.30	<0.001	5.64	4.90	8.15	5.68	−7.19	902.61	<0.001	−0.51
Factor II	12.33	<0.001	3.24	4.17	4.28	4.81	−3.52	903.65	<0.001	−0.21
Factor III	9.94	0.002	9.77	6.10	10.46	6.97	−1.60	905.68	0.108	–
Factor IV	0.04	0.828	11.21	3.85	12.19	3.92	−3.80	919	<0.001	−0.25

*SRAS-R, School Refusal Assessment Scale-Revised; Factor I, to avoid negative affectivity; Factor II, to avoid social aversion and/or evaluation; Factor III, to pursue attention; Factor IV, to pursue tangible reinforcement.*

Differences found between the groups of students having high and low scores on Verbal Aggression, based on the type of school refusal behavior, are presented in Table 3. Results show that students having high scores on Verbal Aggression obtained a higher mean score on school refusal behavior, with statistically significant differences for all of the factors of SRAS-R (I. Avoidance of negative affectivity; II. Escape from aversive social and/or evaluative situations; III. Pursuit of attention from significant others; and IV. Pursuit tangible reinforcement outside of school). The size of the differences found was small for Factor II (*d* = 0.33), Factor III (*d* = 0.16), and Factor IV (*d* = 0.24), while it was medium for Factor I (*d* = 0.54).

**TABLE 3:** Differences in school refusal behavior in students with high and low scores on Verbal Aggression.

**Levene’s test**			**Low score**		**High score**		**Statistics**			
**SRAS-R**	***F***	***p***	***M***	**SD**	***M***	**SD**	***t***	**d.f.**	***p***	***d***
Factor I	10.61	0.001	5.33	4.98	8.26	5.73	−8.10	870.23	<0.001	−0.54
Factor II	14.22	<0.001	2.89	4.25	4.40	4.86	−4.90	869.26	<0.001	−0.33
Factor III	8.08	0.005	9.35	6.26	10.41	6.85	−2.38	858.83	0.017	−0.16
Factor IV	3.70	0.055	11.18	4.04	12.09	3.73	−3.46	880	0.001	−0.24

*SRAS-R, School Refusal Assessment Scale-Revised; Factor I, to avoid negative affectivity; Factor II, to avoid social aversion and/or evaluation; Factor III, to pursue attention; Factor IV, to pursue tangible reinforcement.*

Differences found between the groups of students having high and low scores on Anger, based on the type of school refusal behavior, are presented in Table 4. Results show that students having high scores on Anger obtained a higher mean score on school refusal behavior, with statistically significant differences for all of the factors of SRAS-R (I. Avoidance of negative affectivity, II. Escape from aversive social and/or evaluative situations, III. Pursuit of attention from significant others, and IV. Pursuit tangible reinforcement outside of school). The size of the differences found was small for Factor III (*d* = 0.15) and Factor IV (*d* = 0.14), while it was medium for Factor I (*d* = 0.66) and Factor II (*d* = 0.50).

**TABLE 4:** Differences in school refusal behavior in students with high and low scores on Anger.

**Levene’s test**			**Low score**		**High score**		**Statistics**			
**SRAS-R**	***F***	***p***	***M***	**SD**	***M***	**SD**	***t***	**d.f.**	***p***	***d***
Factor I	44.53	<0.001	5.23	4.41	8.76	6.04	−10.17	903.21	<0.001	−0.66
Factor II	52.93	<0.001	2.56	3.40	4.81	5.28	−7.78	872.61	<0.001	−0.50
Factor III	7.94	0.005	9.50	6.19	10.53	7.04	−2.35	903.79	0.019	−0.15
Factor IV	0.35	0.551	11.47	3.86	12.01	3.96	−2.03	911	0.042	−0.14

*SRAS-R, School Refusal Assessment Scale-Revised; Factor I, to avoid negative affectivity; Factor II, to avoid social aversion and/or evaluation; Factor III, to pursue attention; Factor IV, to pursue tangible reinforcement.*

Differences found between the groups of students having high and low scores on Hostility, based on the type of school refusal behavior, are presented in Table 5. Results show that students having high scores on Hostility obtained a higher mean score on school refusal behavior, with statistically significant differences for all of the factors of SRAS-R (I. Avoidance of negative affectivity, II. Escape from aversive social and/or evaluative situations, III. Pursuit of attention from significant others, and IV. Pursuit tangible reinforcement outside of school). The size of the differences found was small for Factors III (*d* = 0.40) and IV (*d* = 0.16), medium for Factor II (*d* = 0.71), and large for Factor I (*d* = 0.84).

**TABLE 5:** Differences in school refusal behavior in students with high and low scores on Hostility.

**Levene’s test**			**Low score**		**High score**		**Statistics**			
**SRAS-R**	***F***	***p***	***M***	**SD**	***M***	**SD**	***t***	**d.f.**	***p***	***d***
Factor I	51.12	<0.001	4.96	4.48	9.50	6.24	−12.52	812.48	<0.001	−0.84
Factor II	101.50	<0.001	2.30	3.05	5.51	5.60	−10.67	692.83	<0.001	−0.71
Factor III	17.45	<0.001	8.84	5.94	11.14	7.02	−5.30	872.51	<0.001	−0.40
Factor IV	6.19	0.013	11.40	4.18	12.02	3.73	−2.35	886.09	0.019	−0.16

*SRAS-R, School Refusal Assessment Scale-Revised; Factor I, to avoid negative affectivity; Factor II, to avoid social aversion and/or evaluation; Factor III, to pursue attention; Factor IV, to pursue tangible reinforcement.*

### Predictive Capacity of School Refusal on Aggression

Table 6 provides the results of the logistic regression analysis for the probability of receiving high scores on Physical Aggression based on the type of school refusal. The proportion of correctly classified cases ranged from 55.4% of the cases (χ^2^ = 14.38; *p* = 0.001) for the second factor and 61.2% of the cases (χ^2^ = 50.93; *p* ≤ 0.001) for the first factor of the SRAS-R. The values of the OR were higher than 1 for the school refusal models, with the probability of having high Physical Aggression being 1.10 (Factor I), 1.05 (Factor II), and 1.06 (Factor IV) times greater for each point that the scores increased, respectively, on the cited school refusal dimensions.

**TABLE 6:** Logistic regression model for the probability of presenting high Physical Aggression based on the school refusal behavior.

**SRAS-R**	**AQ**		**χ^2^**	***R*^2^**	***B***	**SE**	**Wald**	***p***	**OR**	**I.C. 95%**
Factor I	Correctly classified:	61.2%	50.93	0.07	0.09	0.01	45.76	<0.001	1.10	1.06–1.13
		Constant			−0.61	0.11	16.33	<0.001	0.54	<0.001
Factor II	Correctly classified:	55.7%	12.55	0.02	0.05	0.01	11.86	0.001	1.05	1.02–1.09
		Constant			−0.18	0.08	4.62	0.032	0.83	
Factor IV	Correctly classified:	55.4%	14.38	0.02	0.06	0.01	14.10	<0.001	1.06	1.03–1.10
		Constant			−0.74	0.21	12.32	<0.001	0.47	<0.001

*SRAS-R, School Refusal Assessment Scale-Revised; Factor I, to avoid negative affectivity; Factor II, to avoid social aversion and/or evaluation; Factor IV, to pursue tangible reinforcement.*

Table 7 offers the results of the logistic regression analysis for the probability of receiving high scores on Verbal Aggression based on the type of school refusal. The proportion of correctly classified cases ranged from 56.1% of the cases (χ^2^ = 5.616; *p* = 0.019) for the third factor and 64.2% of the cases (χ^2^ = 63.77; *p* ≤ 0.001) for the first factor of the SRAS-R. The values of the OR were higher than 1 for the school refusal models, with the probability of having high Verbal Aggression being 1.11 (Factor I), 1.08 (Factor II), 1.02 (Factor III), and 1.06 (Factor IV) times greater for each point that the scores increased, respectively, on the cited school refusal dimensions.

**TABLE 7:** Logistic regression model for the probability of presenting high Verbal Aggression based on the school refusal behavior.

**SRAS-R**	**AQ**		**χ^2^**	***R*^2^**	***B***	**SE**	**Wald**	***p***	**OR**	**I.C. 95%**
Factor I	Correctly classified:	64.2%	63.77	0.09	0.10	0.11	54.72	<0.001	1.11	1.08–1.14
		Constant			−0.46	0.11	16.32	<0.001	0.63	
Factor II	Correctly classified:	60.0%	24.37	0.04	0.07	0.11	21.36	<0.001	1.08	1.04–1.12
		Constant			−0.03	0.08	0.14	0.708	0.96	
Factor III	Correctly classified	56.1%	5.61	0.04	0.02	0.01	5.52	0.019	1.02	1.01–1.05
		Constant			0.01	0.12	0.01	0.977	1.01	
Factor IV	Correctly classified:	57.6%	11.92	0.02	0.06	0.01	11.75	0.001	1.06	1.02–1.10
		Constant			−0.45	0.21	4.50	0.034	0.63	

*SRAS-R, School Refusal Assessment Scale-Revised; Factor I, to avoid negative affectivity; Factor II, to avoid social aversion and/or evaluation; Factor III, to pursue attention; Factor IV, to pursue tangible reinforcement.*

Table 8 offers the results of the logistic regression analysis for the probability of receiving high scores on Anger based on the type of school refusal. The proportion of correctly classified cases ranged from 54.3% of the cases (χ^2^ = 4.13; *p* = 0.042) for the fourth factor and 64.1% of the cases (χ^2^ = 95.90; *p* ≤ 0.001) for the first factor of the SRAS-R. The values of the OR were higher than 1 for the school refusal models, with the probability of having high scores on Anger being 1.13 (Factor I), 1.13 (Factor II), 1.02 (Factor III), and 1.04 (Factor IV) times greater for each point that the scores increased, respectively, on the cited school refusal dimensions.

**TABLE 8:** Logistic regression model for the probability of presenting high Anger based on the school refusal behavior.

**SRAS-R**	**AQ**		**χ^2^**	***R*^2^**	***B***	**SE**	**Wald**	***p***	**OR**	**I.C. 95%**
Factor I	Correctly classified:	64.1%	95.90	0.13	0.13	0.01	78.94	<0.001	1.13	1.10–1.17
		Constant			−0.66	0.11	32.86	<0.001	0.51	
Factor II	Correctly classified:	60.6%	59.31	0.08	0.12	0.01	46.71	<0.001	1.13	1.10–1.18
		Constant			−0.23	0.09	6.69	0.010	0.79	
Factor III	Correctly classified	55.4%	5.42	0.01	0.02	0.01	5.34	0.021	1.02	1.01–1.04
		Constant			0.01	0.12	0.01	0.892	0.98	
Factor IV	Correctly classified:	54.3%	4.13	0.01	0.03	0.01	4.11	0.042	1.04	1.01–1.07
		Constant			−0.18	0.21	0.79	0.372	0.82	

*SRAS-R, School Refusal Assessment Scale-Revised; Factor I, to avoid negative affectivity; Factor II, to avoid social aversion and/or evaluation; Factor III, to pursue attention; Factor IV, to pursue tangible reinforcement.*

Table 9 offers the results of the logistic regression analysis for the probability of receiving high scores on Hostility based on the type of school refusal. The proportion of correctly classified cases ranged from 52.7% of the cases (χ^2^ = 5.55; *p* = 0.019) for the fourth factor and 67.6% of the cases (χ^2^ = 148.4; *p* ≤ 0.001) for the first factor of the SRAS-R. The values of the OR were higher than 1 for the school refusal models, with the probability of having high scores on Hostility being 1.17 (Factor I), 1.21 (Factor II), 1.05 (Factor III), and 1.04 (Factor IV) times greater for each point that the scores increased, respectively, on the cited school refusal dimensions.

**TABLE 9:** Logistic regression model for the probability of presenting high Hostility based on the school refusal behavior.

**SRAS-R**	**AQ**		**χ^2^**	***R*^2^**	***B***	**SE**	**Wald**	***p***	**OR**	**I.C. 95%**
Factor I	Correctly classified:	67.6%	148.41	0.20	0.16	0.01	113.05	<0.001	1.17	1.14–1.21
		Constant			−1.12	0.12	84.20	<0.001	0.32	
Factor II	Correctly classified:	65.2%	120.01	0.17	0.19	0.02	83.01	<0.001	1.21	1.17–1.27
		Constant			−0.69	0.09	51.25	<0.001	0.49	<0.001
Factor III	Correctly classified	58.2%	27.83	0.04	0.05	0.01	26.49	<0.001	1.05	1.03–1.08
		Constant			−0.54	0.12	19.36	<0.001	0.57	<0.001
Factor IV	Correctly classified:	52.7%	5.55	0.01	0.04	0.01	5.51	0.019	1.04	1.01–1.07
		Constant			−0.46	0.20	4.85	0.028	1.58	

*SRAS-R, School Refusal Assessment Scale-Revised; Factor I, to avoid negative affectivity; Factor II, to avoid social aversion and/or evaluation; Factor III, to pursue attention; Factor IV, to pursue tangible reinforcement.*

## Discussion

The purpose of this study was to examine the predictive relationships between school refusal behavior and aggression in Spanish adolescents aged 13–17. To achieve this objective, first, the differences in mean scores on school refusal behavior based on the high and low scores on Physical Aggression, Verbal Aggression, Anger, and Hostility were compared. Then, the predictive capacity of school refusal behavior on high scores on aggression was analyzed.

Given that school refusal behavior has been associated with aggressive and criminal attitudes (Heyne and Sauter, 2013), these findings reinforce the idea of an association between these issues. In this work, students with high scores on the distinct types of aggression, in general, were found to have statistically significant higher scores on the four types of school refusal behavior, thereby confirming Hypothesis 1. It should be noted that, until now, most studies analyzing the comorbidity between school refusal behavior from a functional approach and externalizing behavior problems had associated it with the fourth factor of the SRAS-R (Kearney and Albano, 2004). This could be explained by the fact that the fourth factor is not related to an attitude of school refusal based on anxiety or fears associated to the educational contexts (Kearney, 2008; Gonzálvez et al., 2018). The results of this study, however, are novel, given that upon analyzing the specific manifestation of aggression, differentiating between four distinct dimensions (Physical, Verbal, Anger, and Hostility), not only did those students that based their school refusal on the fourth factor receive higher scores, but the other causes of refusal behavior were also associated with aggressive behavior. This may be due to the fact students with high scores on school refusal, based on the first three SRAS-R factors, have also been associated with internalizing behaviors. In other words, they display manifestations of anxious and depressive behavior (Evren et al., 2015; Finning et al., 2019; Knollmann et al., 2019; Lawrence et al., 2019; Fornander and Kearney, 2020). These behaviors, characterized by a low emotional control, may lead to the manifestation of aggressive behavior as a means of escape (Henry et al., 2012; Bucur et al., 2020; Fernández-Sogorb et al., 2020). According to Law et al. (2011), how an individual behaves in the face of distinct situations may have repercussions on the manifestation of emotions such as anger or aggression, thus increasing the risk of impulsive or aggressive behavior that may lead to school refusal (Shaikh et al., 2019). Therefore, emotionally vulnerable students could react with aggressive-based responses to cope with stressful school situations (Torregrosa et al., 2020). As for the predictive analysis, the results confirm Hypothesis 2, with school refusal acting as a positive and statistically significant predictor of aggression. The relationship between both variables suggests that, in most cases, the distinct causes of school refusal act as predictors of high aggression scores. These results coincide with those of Aparicio-Flores et al. (2020), who considered a Spanish early childhood-aged sample and found a positive predictive relationship between school refusal and high levels of aggressive behavior.

Despite the contributions of this study, certain limitations should also be considered. First, the limited number of documents found on the subject prevented a contrast of the results for distinct samples. Therefore, future studies should examine the relationship between these two variables in order to offer greater validity and consistency to these study results. Due to the cross-sectional nature of the study design, causal inferences cannot be made, and it is impossible to generalize the findings to other ages and cultures. It would be interesting to examine this relationship in samples from other countries and in an early childhood-aged sample, given the importance of early intervention to reduce subsequent absenteeism and school dropout during adolescence (Escobar et al., 2016; Gonzálvez et al., 2018), and longitudinal studies would be useful to establish causal relationships between the analyzed variables. Although our findings are with adolescents, aggressive behavior can be developed from an early age (Hay, 2017). According to previous studies, while physical aggression decreases from childhood to adolescence, social aggression increases (Kikas et al., 2009). Finally, collecting information about school attendance records and using additional sources of data (student performance, parents’, and teachers’ views) should be considered in future studies.

## Conclusion

To conclude, despite the previously mentioned limitations, this study reinforces the relationship existing between two problems affecting our current education system: aggressive behavior and school attendance problems. A significant relationship was revealed to exist between both variables. Future studies should consider the need to design and offer educational strategies with specific intervention and prevention measures for use by schools and education specialists (Bucur et al., 2020). Preventive interventions could be focused on improving the affective levels of all students, especially the most emotionally vulnerable, which would be positively reflected in the development and happiness of the students due to the relationship found between the school refusal behavior, affectivity, and aggression (Sanmartín et al., 2018; Vicent et al., 2018). Designing these proposals, the different reasons for school refusal must be taken into account in order to offer a more adjusted intervention. In most cases, school refusal behavior was found to be a positive and statistically significant predictor of aggression. However, special attention should be given to those students who base their school refusal by negative reinforcement (Factors I and II) due to its higher prediction scores.

## Data Availability Statement

The raw data supporting the conclusions of this article will be made available by the authors, without undue reservation.

## Ethics Statement

The studies involving human participants were reviewed and approved by the Ethics Committee of the University of Alicante (UA-2017-09-05). Written informed consent to participate in this study was provided by the participants’ legal guardian/next of kin.

## Author Contributions

CG and MM: conceptualization and writing—original draft preparation. RS and MV: methodology and formal analysis. CG, MM, and MV: investigation. RS: writing—review and editing. All authors contributed equally to the research design, data analysis, and revision, and approved the final manuscript.

## Conflict of Interest

The authors declare that the research was conducted in the absence of any commercial or financial relationships that could be construed as a potential conflict of interest.
